# The Role of Vitamin D in Primary Biliary Cirrhosis: Possible Genetic and Cell Signaling Mechanisms

**DOI:** 10.1155/2013/602321

**Published:** 2013-03-26

**Authors:** Khanh vinh quốc Lương, Lan Thi Hoàng Nguyễn

**Affiliations:** Vietnamese American Medical Research Foundation, 14971 Brookhurst Street, Westminster, CA 92683, USA

## Abstract

Primary biliary cirrhosis (PBC) is an immune-mediated chronic inflammatory disease of the liver of unknown etiology. Vitamin D deficiency is highly prevalent in patients with PBC, and many studies have demonstrated the significant effect of calcitriol on liver cell physiology. Vitamin D has antiproliferative and antifibrotic effects on liver fibrosis. Genetic studies have provided an opportunity to determine which proteins link vitamin D to PBC pathology (e.g., the major histocompatibility complex class II molecules, the vitamin D receptor, toll-like receptors, apolipoprotein E, *Nramp1*, and cytotoxic T lymphocyte antigen-4). Vitamin D also exerts its effect on PBC through cell signaling mechanisms, that is, matrix metalloproteinases, prostaglandins, reactive oxygen species, and the transforming growth factor betas. In conclusion, vitamin D may have a beneficial role in the treatment of PBC. The best form of vitamin D for use in the PBC is calcitriol because it is the active form of vitamin D_3_ metabolite, and its receptors are present in the sinusoidal endothelial cells, Kupffer cells, and stellate cells of normal livers, as well as in the biliary cell line.

## 1. Introduction

Primary biliary cirrhosis (PBC) is an immune-mediated chronic inflammatory disease of the liver of unknown etiology, and its progressive destruction of the bile duct leads to fibrosis and liver cirrhosis. There is evidence of aberrations in the vitamin D-endocrine system in PBC patients. Skeletal demineralization and low serum concentrations of 25-hydroxyvitamin D_3_ (25OHD) were observed in patients with PBC [1–4]. Vitamin-D absorption was reduced in PBC patients and correlated with the amount of fecal fat [5–7]. Many studies have shown a significant effect of calcitriol on liver cell physiology. Calcitriol increases intracellular Ca^2+^ in rat hepatocytes [8] and controls DNA polymerase *α* activity, as well as cytoplasmic and nuclear protein kinase activity, promoting normal liver recovery after partial hepatectomy in rats [9]. Vitamin D was also shown to have a detoxifying effect in human primary cultured hepatocytes by increasing the expression of P_450_ cytochromes (i.e., CYP3A4, CYP2B6, and CYP2C9) [10]. Some studies failed to detect VDR levels in the liver [11, 12]; however, Gascon-Barré et al. [13] demonstrated that human, rat, and mouse hepatocytes express very low nuclear vitamin D receptor (nVDR) mRNA and protein levels. In contrast, the sinusoidal endothelial, Kupffer, and stellate cells of normal livers, as well as the biliary cell line and rat hepatic neonatal epithelial cells, clearly expressed both nVDR mRNA and protein. Berger et al. [14] demonstrated that calcitriol receptors were localized in the nucleus and widely distributed in normal human tissues, including those of the liver, kidney, thyroid, adrenal glands, gastrointestinal tract, breast, and skin. The calcitriol-binding proteins were present in liver nuclei isolated from mice, rabbits, chickens, and cultured rat hepatocytes [15]. A major metabolite of the vitamin D analog 1*α*-hydroxyvitamin D_2_, 1*α*,24(S)-hydroxyvitamin D_2_, was identified in human liver cells in culture and binds strongly to the VDR [16]. Another report demonstrated the presence of VDR mRNA and protein in the livers of rats during all periods of the rats' lives [17]. Furthermore, VDR ligands have the potential to prevent a cholestasis-induced inflammatory response. In a mouse model of cholestasis, calcitriol treatment altered the expression of the genes involved in bile acid synthesis and transport in the liver and also suppressed the mRNA expression of proinflammatory cytokines in the liver resulting in decreased plasma levels of proinflammatory cytokines [18]. It has been suggested that proliferating cholangiocytes play a key role in chronic cholestasis liver diseases that are characterized by biliary fibrosis [19]. Calcitriol protects liver cells during cholestasis by inhibiting CYP7A1 mRNA expression and bile acid synthesis [20], and both *in vitro* and *in vivo* models have demonstrated calcitriol's antiproliferative and antifibrotic effects on liver fibrosis [21]. Another link of PBC to vitamin D may be the observation that PBC has seasonal variation; there was a marked peak for diagnoses of PBC in the month of June [22]. Furthermore, high prevalence for fatigue was reported in PBC patients. Al-Harthy et al. [23] demonstrated that fatigue appeared to have improved fatigue score in PBC patients taking calcium and vitamin D, whereas biochemical response to ursodeoxycholic acid (UDCA) treatment was not associated with lower fatigue score. These findings suggest that vitamin D may have a role in PBC. Therefore, we will discuss the role of vitamin D in PBC with possible genetic and cell signaling mechanisms.

## 2. Genetic Factors Related to Vitamin D in Primary Biliary Cirrhosis

Studies have suggested that several genes in the major histocompatibility complex (MHC) region promote susceptibility to PBC. Located in the MHC region, human leukocyte antigen (HLA) genes have been implicated in PBC susceptibility. A significant *DRw8* allele was found to be increased in patients with PBC compared with controls [24], and the DRB1*-0801-DQA1*0401-DQB1*0402 haplotype was a marker of disease progression but not of initial susceptibility [25]. A strong association between PBC and the DPB1*0301 allele was found in a German population [26]. Although an association between the DPB1*0501 allele and PBC was demonstrated in Japanese patients [27], this association was not observed in the German population [26]; this discrepancy is due to differences in the distribution of *DPB1* alleles in both ethnic groups [28]. An association with the DRB1*08 allele was found in German, Spanish, Swedish, and American populations with PBC [29], whereas the reported association with PBC is with DRB1*0803 in the Japanese population [30]. Significant associations were found with PBC susceptibility for the DRB1*0803-DQB1*0601 and DRB1*0405-DQB1*0401 haplotypes; in contrast, significant protective associations were found between PBC and the DRB1*1302-DQB1*0604 haplotype and DRB1*1101-DQB1*0301 haplotypes in the Japanese population [31]. These data show that there are HLA class II alleles associated with susceptibility to and protection from PBC and that these differ between ethnic groups. Calcitriol is known to stimulate phagocytosis but suppresses MHC class II antigen expression in human mononuclear phagocytes [32, 33]. Calcitriol also decreases interferon-gamma-induced HLA-DR antigen expression in normal and transformed human keratinocytes [34, 35]. These findings suggest that calcitriol may have an effect on PBC through suppressing the expression of MHC class II antigens.

Genetic studies provide an opportunity to link molecular variations with epidemiological data. DNA sequence variations, such as polymorphisms, exert both modest and subtle biological effects on the molecules. Vitamin D exhibits immunomodulatory and antiproliferative effects through the VDR in diseases. There is an association between *BsmI* polymorphisms of the VDR and PBC in German, Hungarian, Japanese, Italian, and Chinese patients [36–40]. The *BB* genotype contributes to the risk of PBC development. In the Polish population, *BsmI* and *TaqI* polymorphisms were associated with the presence of advanced fibrosis and liver cirrhosis with the diagnosis of PBC [41]. In addition, *VDR* gene polymorphisms were found to be associated with osteoporosis, in which *VDR* polymorphisms were predictive of decreased bone mineral density in PBC patients [42, 43]. These reports suggested that alterations in VDR function may play a role in PBC.

Toll-like receptors (TLRs) are a group of glycoproteins that function as surface transmembrane receptors and are involved in the innate immune responses to exogenous pathogenic microorganisms. Substantial evidence points to the importance of TLRs in the pathogenesis and outcome of PBC. Monocytes from PBC patients appear more sensitive to signaling via select TLRs, resulting in the secretion of selective proinflammatory cytokines [44]. The expression of TLR-3 was markedly increased in biliary epithelial cells (BECs) at ductular reaction sites in liver diseases, including PBC [45]. TLR-3 and type I interferon (IFN) signaling pathways are activated in both the portal tract and parenchyma in early-stage PBC [46]. In addition, the bile duct epithelial cells of PBC liver tissues markedly expressed TLR-4, which was also observed in the periportal hepatocytes of PBC liver tissues, and its expression extended to interlobular hepatocytes in advanced-stage PBC [47]. The expression of TLR-4 and the activation of the natural killer NF-*κ*B transcription factor were significantly enhanced in the liver tissues of PBC patients [48, 49] and destroyed autologous BECs in the presence of INF-*α* synthesized by TLR-3 ligand-stimulated monocytes [50]. Furthermore, the CpG motif induces the secretion of antimitochondrial antibodies in peripheral blood mononuclear cells and also upregulates the B-cell expression of TLR-9 in patients with PBC [51]. The *2848 AA TLR-9* genotype influences the immune response to the CpG motif and contributes to hyper-IgM syndrome in PCB [52]. Vitamin D deficiency increases the expression of hepatic mRNA levels of TLR-2, TLR-4, and TLR-9 in obese rats [53]; however, calcitriol suppresses the expression of TLR-2 and TLR-4 protein and mRNA in human monocytes and triggers hyporesponsiveness to pathogen-associated molecular patterns [54]. Calcitriol has also been shown to downregulate intracellular TLR-2, TLR-4, and TLR-9 expression in human monocytes [55]. TLR activation results in the expression of the VDR and 1*α*-vitamin D hydroxylase in human monocytes [56]. In addition, calcitriol can increase the vitamin-D-induced expression of cathelicidin in bronchial epithelial cells [57] and may enhance the production of cathelicidin LL-37 [58]. The addition of a VDR antagonist has been shown to inhibit the induction of cathelicidin mRNA by more than 80%, which consequently reduces the protein expression of this antimicrobial agent by approximately 70% [57]. Biliary epithelial cells show intense immunereactivity to cathelicidin and to the VDR. In cultured biliary epithelial cells, endogenous bile salt chenodeoxycholic acid and therapeutic bile salt UDCA induce cathelicidin expression through two different nuclear receptors, such as the farnesoid X receptor and VDR [59]. These findings indicate that bile salt may contribute to biliary tract sterility by controlling epithelial cell innate immunity and combining bile salt with vitamin D which would increase therapeutic efficacy in inflammatory biliary disease. Taken together, vitamin D may have a role in PBC patients by modifying TLR pathways.

Apolipoprotein E (ApoE) has important functions in systemic and local lipid transport. In PBC, serum ApoE was increased and partly contributed to the decrease in the levels of hepatic triglyceride lipase [60, 61]. In addition, a close relationship existed between the ratio of ApoE to Apo A_1_ and plasma bile salt concentration [62]. The *ApoE *ε*4* allele has been suggested to be a marker of disease severity in PBC [63]. At the time of diagnosis of PBC, the *ε*4 allele carriers were younger, had higher bilirubin and IgG levels, and had a lower prothrombin index compared with *ε*2 or *ε*3 homozygous allele carriers. The *ε*4 allele carriers demonstrated a poor response to UDCA treatment. However, the frequency of the *ε*2 allele is overexpressed in patients with PBC and did not respond to UDCA treatment compared with *ε*4 allele carriers in the Finnish population [64]. In contrast, the *ApoE4* allele is reported to be associated with decreased bone mass in postmenopausal Japanese women [65]. The common *ApoE* polymorphism has a complex effect on bone metabolism in peri-menopausal Danish women; namely, those with *ApoE2* have a lower rate of bone mineral loss in the femoral neck and hip regions compared with other women, whereas those with *ApoE4* gain more bone mineral than other women [66]. Calcitriol has been shown to induce macrophages to exhibit specific saturable receptors for low-density lipoprotein (LDL), and acetyl LDL, the LDL receptor of 1,25OHD-induced macrophages, has been found to exhibit specificity for ApoB- and ApoE-containing lipoproteins [67]. In ApoE knockout mice, those with dyslipidemia, high oxidative stress, and pronounced atherosclerosis after unilateral nephrectomy developed less plaque growth and calcification with vitamin D analog treatment (paricalcitol) compared with healthy controls [68, 69]. ApoE *ε*4, however, is associated with higher serum 25OHD levels [70]. These findings suggested that vitamin D may improve lipid profiles and cholesterol metabolisms in PBC patients.

*Nramp1* plays a critical role in macrophage defenses against intracellular pathogens. The expression of *Nramp1* in pathogen-containing phagosomes is associated with enhanced fusion to lysosomes, increased phagosomal acidification, and greater bactericidal activity. The *NRAMP1* gene has been shown to regulate the concentration of divalent cations in the phagosomes of macrophages [71], and its antibacterial role may be a result of the extrusion of protons and divalent metal ions from the phagosomal lumen toward the cytoplasm [72]. PMNs are the major site of *NRAMP1* expression, followed to a lesser degree by monocytes [73]. The *Nramp1* gene has been identified among inbred mouse strains as a factor for host defense against some species of mycobacteria [74]. Moreover, antibodies to a 65 kDa mycobacterial protein were found in patients with PBC [75, 76]. Graham et al. [77] identified novel alleles at a polymorphic microsatellite repeat region in the human *NRAMP1* gene promoter in patients with PBC. However, calcitriol is known to stimulate phagocytosis [32] and affects *NRAMP1* transcription and protein expression in maturing phagocytes [78]. Taken together, vitamin D may have a role in PBC patients by modifying *NRAMP1* transcription and protein expression.

Cytotoxic T lymphocyte antigen-4 (CTLA-4) is involved in the regulation of T cells and is a coregulatory immuno-receptor that has a broad dampening effect on T-cell responses [79]. The PBC is known to be associated with polymorphisms of the CTLA-4 gene in Chinese [80], Japanese [81], Canadian [82], French [83], America Caucasian [84], and British [85] patients, but not in Brazilian patients [86]. The meta-analysis results suggested that the CTLA-4 gene may be a risk factor for PBC in Asians [87–89], whereas the AA genotype may have negative associations with PBC in Asians [89, 90]. A deficiency of CTLA-4 causes a severe lymphoproliferative disorder and demonstrates multiorgan autoimmunity, leading to massive tissue destruction and early death [91, 92]. Furthermore, CTLA-4 Ig suppressed a lupus-like illness in mouse models [93]. Nonobese diabetic (NOD) mice treated with CTLA-4 Ig after the onset of insulitis had a reduced incidence of diabetes [94]. However, calcitriol promoted regulatory T-cell profiles by increasing CTLA-4 and interleukin-10 in mouse colon protein extracts [95]. Calcitriol also stimulated the expression of high levels of CTLA-4 in human CD4^+^ CD25^−^ T cells [96]. These findings suggested that vitamin D may have a role in PBC patients by enhancing CTLA-4 levels.

Table 1 summarizes the genetic factors associated with vitamin D and PBC.

## 3. The Cell Signaling Mechanisms to Vitamin D in Primary Biliary Cirrhosis

Matrix metalloproteinases (MMPs) are proteolytic enzymes that are responsible for extracellular matrix remodeling and the regulation of leukocyte migration through the extracellular matrix, which is an important factor involved in inflammatory processes and infectious diseases. MMPs are produced by many cell types including lymphocytes, granulocytes, astrocytes, and activated macrophages. Mdr2^−/−^mice develop hepatic lesions resembling primary sclerosing cholangitis and spontaneously progress to severe fibrosis, accompanied by the upregulation of MMP-2, MMP-13, and TIMP-1 [97]. Increased serum MMP-1 and -2 were observed in patients with PBC [98–100]. Polymorphism of MMP-3 influences susceptibility to primary sclerosing cholangitis [101]. However, VDR knockout mice had an increased influx of inflammatory cells, phosphoacetylation of NF-*κ*B associated with increased proinflammatory cells, and upregulation of MMP-2, MMP-9, and MMP-12 mRNA and enzyme levels in the lung [102]. The *VDR TaqI* polymorphism is associated with decreased production of TIMP-1, which is a natural inhibitor of MMP-9 [103]. Calcitriol modulates tissue MMP expression under experimental conditions [104], downregulates MMP-9 levels in keratinocytes, and may attenuate the deleterious effects caused by the excessive TNF-*α*-induced proteolytic activity associated with cutaneous inflammation [105]. Calcitriol inhibits both basal and staphylococcus-stimulated production of MMP-9 in human blood monocytes and alveolar macrophages [106]. Moreover, a vitamin D analog was reported to reduce the expression of MMP-2, MMP-9, VEGF, and PTH-related peptide in Lewis lung carcinoma cells [107]. Calcitriol significantly attenuated *M. tuberculosis*-induced increases in the expression of MMP-7 and MMP-10 and suppressed the secretion of MMP-7 by *M. tuberculosis-*infected PBMCs. MMP-9 gene expression, secretion, and activity were significantly inhibited, irrespective of infection status [108]. In another study, calcitriol suppressed the production of MMPs (MMP-7 and MMP-9) and enhanced the level of TIMP-1 in tuberculosis patients [109]. In another study, the antifibrotic effect of calcitriol was demonstrated by upregulation of MMP-9 enzyme activity, but not the promoter level in HSC [21]. However, vitamin D promotes both up- and downregulation of MMP activity, depending on the specific cell type. These studies suggest that calcitriol may play an important role in the pathological process of PBC by regulating the level of MMPs and TIMPs. 

Prostaglandins (PGs) play a role in inflammatory processes [110]. Cyclooxygenase (COX) participates in the conversion of arachidonic acid into PGs. In chronic bile duct ligation, there is an increase in PG E_2_ excretion [111]. Phytohemagglutinin-(PHA-) stimulated enriched monocytes from PBC patients produced approximately threefold more PG E_2_ than did normal control monocytes and alcoholic cirrhosis monocytes [112]. The monocyte-produced PG E_2_ may be responsible for the hyporesponsiveness to PHA observed in PBC patients [113]. UDCA normalized the defective natural killer activity in PBC by inhibiting PG E_2_ production [114]. Moreover, PBC epithelial cells have shown moderate levels of COX-2 expression [115]. Calcitriol has been reported to regulate the expression of several key genes involved in PG pathways, causing a decrease in PG synthesis [116]. Calcitriol and its analogs have also been shown to selectively inhibit the activity of COX-2 [117]; namely, the VDR agonist, elocalcitol, decreased COX-2 expression and PG E_2_ production in benign prostatic hyperplasia cells [118]. These findings suggested that vitamin D may play a role in modulating the inflammatory process in PBC.

Reactive oxygen species (ROS) have been suggested to play a role in PBC. Lipid peroxidation markers, such as plasma and urinary 8-isoprostane and plasma malondialdehyde (MDA), were significantly increased, whereas plasma total glutathione (GSH) levels were reduced in patients with PBC [119]. UDCA treatment partially corrected plasma GSH status in patients with PBC, and it also decreased portal pressure and increased levels of hepatic GSH and superoxide dismutase (SOD) activity in bile duct-ligated rats [120, 121]. Calcitriol has been reported to exert a receptor-mediated effect on the secretion of hydrogen peroxide by human monocytes [122]. Human monocytes in culture gradually lose their capability to produce superoxide when stimulated; the addition of calcitriol, lipopolysaccharide, or lipoteichoic acid (LTA) restored the ability of stimulated monocytes to produce superoxide and increased oxidative capacity compared with unstimulated monocytes [123]. Calcitriol may also protect nonmalignant prostate cells from oxidative stress-induced cell death by eliminating ROS-induced cellular injuries [124]. Vitamin D metabolites and vitamin D analogs were reported to induce lipoxygenase mRNA expression, lipoxygenase activity, and ROS production in a human bone cell line [125]. Vitamin D may reduce the extent of lipid peroxidation and induce SOD activity of the hepatic antioxidant system in rats [126, 127]. In addition, calcitriol also enhances intracellular GSH pools and significantly reduces nitrite production induced by lipopolysaccharides (LPS) [128]. These findings suggested that vitamin D modulates oxidative stress in PBC.

The transforming growth factor-betas (TGF-*β*s) are a group of homologous polypeptides that exert pleiotropic effects on various cell types and stimulate extracellular matrix formation and fibrosis. Northern blot analysis has revealed enhanced expression of TGF-*β*_1_, TGF-*β*_2_, TGF-*β*_3_, and their receptors in human liver cirrhosis [129]; TGF-*β*_2_ has been identified as an actively transcribed TGF-*β* gene during the progression of liver fibrosis in biliary atresia [130]; statistically significant changes in serum TGF-*β*_1_ levels have been demonstrated in common bile duct-ligated rats and in PBC patients [131–133]; an overexpression of TGF-*β*_3_ has been observed in hepatocytes in PBC patients [134]. However, TGF-*β* has been detected in the majority of specimens from patients with PBC [135]; and a marker of TGF-*β* pathway signaling has been identified in the biliary endothelial cells of patients with posttransplantation recurrence of PBC [136]. Furthermore, TGF-*β* levels have been reported to be higher in the bone marrow mononuclear cells of PBC patients compared with autoimmune hepatitis type 1 patients [137, 138]. UDCA treatment has reduced TGF-*β* levels in patients with PBC [139]. The inhibition of *α*v*β*6 potently inhibits the progression of primary and secondary biliary fibrosis and also blocks TGF-*β* activation [140]. On the other hand, TGF-*β* levels have correlated negatively with vitamin D levels; namely, increased TGF-*β*_1_ and platelet counts have been an early indicator of bone marrow fibrosis in patients with vitamin D deficiency [141]. An association has been found between the *FokI* VDR polymorphism and plasma concentrations of TGF-*β* in patients with type 1 diabetes mellitus [142]. Among diabetic children, higher levels of TGF-*β*_1_ were observed compared with healthy children, and the diabetic carriers of the *ff* genotype have shown low levels of 25OHD compared with the carriers of the *F* allele. Vitamin D has a significant effect on regulating levels of bioactive TGF-*β*_1_ and appears to affect aspects of the TGF-*β*_1_ signaling pathway in renal tissue [143]. Calcitriol reduces TGF-*β*_3_-induced fibrosis-related gene expression in human leiomyoma cells [144]. Moreover, vitamin D treatment significantly downregulates the free fatty acid-induced expression of TGF-*β* in HSC line LX-2 [145]. These results suggest that vitamin D may participate in the reduction of inflammatory and profibrogenic activity in PBC. 

Table 2 summarizes the cell signaling mechanisms to vitamin D in PBC.

## 4. Conclusion

The relationship between vitamin D and PBC has been discussed. Vitamin D may have a beneficial role in PBC and has been demonstrated to have antiproliferative and antifibrotic effects on liver fibrosis. Genetic studies have provided an opportunity to determine which proteins link vitamin D to PBC pathology. Vitamin D also exerts its effect on PBC through nongenomic mechanisms. The best form of vitamin D for use in the treatment of PBC is calcitriol because it contains the active form of the vitamin D_3_ metabolite and its receptor is present in the sinusoidal endothelial, Kupffer, and stellate cells of normal livers, as well as in the biliary cell line.

## Figures and Tables

**Table 1:** Genetic factors related to vitamin D and primary biliary cirrhosis.

	Primary biliary cirrhosis (PBC)	Vitamin D
Human leukocyte antigen (HLA)	HLA-DR expression increased in patients with PBC [24–31]	Calcitriol suppresses MHC class II antigen expression in human mononuclear phagocytes and decreases interferon-*γ*-induced HLA-DR antigen expression in normal and transformed human keratinocytes [32–35]

Vitamin D receptor (VDR)	(i) *BsmI* polymorphisms of VDR were associated with PBC in German, Hungarian, Japanese, Italian, and Chinese patients [36–40]
	(ii) In the Polish population, *BsmI* and *TaqI* polymorphisms were associated with the presence of advanced fibrosis/liver cirrhosis at the diagnosis of PBC [41]
	(iii) VDR polymorphisms predicted a decreased bone mineral density in PBC patients [42, 43]

Toll-like receptors (TLRs)	(i) The expression of TLR-3 was markedly increased in biliary epithelial cells (BECs) in areas of ductular reaction in liver diseases, including PBC [45]	(i) Calcitriol has been shown to down-regulate intracellular TLR-2, TLR-4, and TLR-9 expression in human monocytes [55]
	(ii) The bile duct epithelial cells in PBC liver tissues markedly expressed TLR-4, which was also observed in periportal hepatocytes of PBC liver tissues and its expression was extended to interlobular hepatocytes in advanced-stage PBC [47]	(ii) TLR activation results in the expression of the VDR and 1*α*-vitamin D hydroxylase in human monocytes [56]
		(iii) Biliary epithelial cells show intense immune reactivity for cathelicidin and for VDR [59]

Apolipoprotein E (ApoE)	(i) The ApoE *ε*4 allele was suggested as a marker of disease severity in PBC [63]	(i) The LDL receptor on calcitriol-induced macrophages has been found to exhibit specificity for ApoB- and ApoE-containing lipoproteins [67]
	(ii) The *ε*4 allele carriers demonstrated a poor response to ursodeoxycholic acid (UDCA) treatment [64]	(ii) Among ApoE knockout mice, those with dyslipidemia, high oxidative stress, and pronounced atherosclerosis after unilateral nephrectomy developed less plaque growth and calcification with vitamin D analog treatment (paricalcitol) [60–68]

Nramp1	Novel alleles at a polymorphic microsatellite repeat region in the human *NRAMP1* gene promoter were identified in patients with PBC [32]	Calcitriol is known to stimulate phagocytosis and affects *NRAMP1* transcription and protein expression in maturing phagocytes [78]

Cytotoxic T lymphocyte antigen-4 (CTLA-4)	(i) PBC is known to be associated with polymorphisms of the CTLA-4 gene in Chinese, Japanese, Canadian, French, American Caucasian, and British patients but not in Brazilian patients [80–86] (ii) The meta-analysis suggests that the CTLA-4 gene may be a risk factor for PBC in Asians, while the AA genotype may be negatively associated with PBC [87–89]	Calcitriol promoted regulatory T-cell profiles by increasing CTLA-4 and interleukin-10 in mouse colon protein extracts and stimulated the expression of high levels of CTLA-4 in human CD4^+^ CD25^−^ T cells [95, 96]

**Table 2:** Summary of the cell signaling mechanisms to Vitamin D primary biliary cirrhosis.

	Primary biliary cirrhosis	Vitamin D
The matrix metalloproteinases (MMPs)	(i) Mdr2^−/−^ mice develop hepatic lesions resembling primary sclerosing cholangitis and spontaneously progress to severe fibrosis accompanied by the upregulation of MMP-2, MMP-13, and TIMP-1 [97]	(i) VDR knockout mice had increased MMP-2, MMP-9, and MMP-12 in the lung [102]
	(ii) Increased serum MMP-1 and -2 were observed in patients with PBC [98–100]	(ii) The *VDR TaqI* polymorphism is associated with the decreased production of TIMP-1 [103]
	(iii) Polymorphism of MMP-3 influences susceptibility to primary sclerosing cholangitis [101]	(iii) Calcitriol modulates tissue MMP expression under experimental conditions and downregulates MMP-9 levels in keratinocytes [104]

Prostaglandins (PGs)	(i) Phytohemagglutinin-stimulated enriched monocytes from PBC patients produced approximately threefold more PG E_2_ than did normal control monocytes and alcoholic cirrhosis monocytes [112] (ii) PBC epithelial cells showed moderate levels of COX-2 expression [115]	Calcitriol regulates the expression of several key genes involved in PG pathways [116]

Reactive oxygen species (ROS)	(i) Lipid peroxidation markers, such as plasma and urinary 8-isoprostane and plasma malondialdehyde (MDA), were significantly increased, whereas plasma total glutathione (GSH) levels reduced in patients with PBC [119]	Vitamin D reduces ROS [123–128]
	(ii) Ursodeoxycholic acid (UCDA) treatment partially corrected plasma GSH status in patients with PBC [120]	

Transforming growth factor *β* (TGF-*β*)	(i) TGF-*β*_3_ was overexpressed in hepatocytes in PBC patients [133] (ii) TGF-*β* pathway signaling was identified in the biliary endothelial cells of patients with posttransplantation recurrence of PBC [136] (iii) TGF-*β* levels were also reported to increase in the bone marrow mononuclear cells of PBC patients compared with autoimmune hepatitis type 1 patients [137, 138] (iv) UCDA treatment reduced TGF-*β* levels in patients with PBC [139]	(i) TGF-*β* levels correlated negatively with vitamin D levels; namely, increased TGF-*β*_1_ and platelet counts are an early indicator of bone marrow fibrosis in patients with vitamin D deficiency [141] (ii) Calcitriol reduces TGF-*β*_3_-induced fibrosis-related gene expression in human leiomyoma cells [144] (iii) Vitamin D treatment significantly down-regulated the free fatty acids-induced expression of TGF-*β* in HSC line LX-2 [145]
